# Hepatitis A among refugees, asylum seekers and migrants living in hosting facilities, Greece, April to December 2016

**DOI:** 10.2807/1560-7917.ES.2017.22.4.30448

**Published:** 2017-01-26

**Authors:** Kassiani Mellou, Anthi Chrisostomou, Theologia Sideroglou, Theano Georgakopoulou, Maria Kyritsi, Christos Hadjichristodoulou, Sotirios Tsiodras

**Affiliations:** 1 Hellenic Centre for Disease Control and Prevention (HCDCP), Athens, Greece; 2 University of Thessaly, Medical School, Larisa, Greece; 3 National and Kapodistrian University of Athens, Medical School, Athens, Greece

## Abstract

An increased number of hepatitis A cases among refugees, asylum seekers and migrants residing in hosting facilities in Greece were recorded between April and December 2016. In total, 177 laboratory-confirmed symptomatic cases were reported; of these, 149 (84%) occurred in hosting camps mostly among Syrian children under 15 years. All cases reported symptom onset after their entry into the country. Public health interventions focused on hygiene measures and vaccination.

In this report, we present the epidemiological data for hepatitis A (HA) cases among refugees, asylum seekers and migrants in hosting facilities in Greece between April and December 2016. We also describe the public health response, the main challenges in the management of the cases and the data from the most affected hosting facilities. For the purpose of this manuscript, we refer to refugees, asylum seekers and migrants, as refugees.

## Case definition

A HA case was defined as any symptomatic case of acute illness with a discrete onset of any sign or symptom, consistent with acute viral hepatitis (e.g. fever, headache, malaise, anorexia, nausea, vomiting, diarrhoea, and abdominal pain), and either (i) jaundice, or (ii) elevated serum alanine aminotransferase (ALT or SGPT) or aspartate aminotransferase (AST or SGOT) levels, and (iii) confirmed by testing for anti-IgM hepatitis A virus (HAV), from 1 April to 31 December 2016, in the population of refugees residing at hosting facilities (camps and others) in Greece.

## Information on data collection

Any HA case presenting at a local health facility was reported to the Hellenic Centre for Disease Control and Prevention (HCDCP) and recorded in a specific database kept by the Department of Epidemiological Surveillance and Response of the HCDCP.

To calculate notification rates, we used as denominators the information available on population estimates: (i) the mean total population residing in hosting facilities between 1 April 2016 and 31 December 2016, according to daily official estimates [1]; (ii) the distribution of the population by age group and country of origin recorded at preregistration of 28,000 refugees between June and July 2016 [2]. We assumed this distribution of population by age group and country of origin to apply to all refugees residing in hosting facilities during the reporting period. The estimates derived were compatible with information on arrivals to Greece [3].

## Laboratory tests and molecular typing

Blood samples from clinically-suspected cases were confirmed by testing for anti-IgM HAV with the locally available method (usually an ELISA test).

The Regional Laboratory of Public Health of Thessaly analysed seven stool samples. All had been collected from Syrian children, aged from 4 to 9 years, and the infection was confirmed by serology. Viral nucleic acids were extracted with the iPrep Invitrogen device using the iPrep Virus Kit. We performed molecular detection and typing of the VP1–2A region of the virus according to the HAV NET typing protocol [4]. The initial reverse transcriptase PCR was performed with the SuperScript One-Step RT-PCR System with Platinum Taq DNA Polymerase, Invitrogen, whereas the nested PCR was performed with KAPA Taq HotStart PCR kit, Kapa Biosystems, according to manufacturers’ instructions. Sequencing followed to an ABI 3730xl Analyser.

## Results

### Epidemiological investigation

In total, 177 HA cases were recorded from 1 April to 31 December 2016.

Cases were reported in 29 different locations: 16 hosting camps (149 cases), 10 hotels (23 cases) and three apartments (5 cases). Of these, 150 cases were hospitalised (85%) after referral by the medical services in the hosting facilities. Triage, laboratory investigation and hospital care were provided free of charge to all cases by the National Health Care System hospitals in Greece.

One hundred and forty two (80%) of the recorded cases presented with jaundice; the average alanine and aspartate aminotransferase values (norm: 7 to 56 and 5 to 40 units per liter (IU/L), respectively) were 1,294 IU/L (standard deviation (SD): 799) and 1,085 IU/L (SD: 866). All cases fully recovered; no complications or cases of fulminant HA or acute liver failure were recorded.

The distribution of notified cases by week of symptom onset is presented in the Figure.

**Figure:**
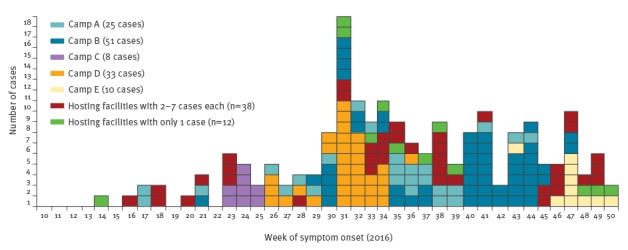
Cases of hepatitis A among refugees by week of symptom onset, Greece, April–December, 2016 (n=177)

All cases reported onset of symptoms at least 50 days after their entry in the country (i.e. after the maximum incubation period for HA). Ninety-six cases were male (54%), the median age of cases was 7 years (range: 8 months–29 years), and 86% (n=152) were children under 15 years.

The majority of cases were from Syria (152 cases) followed by Iraq (9 cases), Afghanistan (8 cases), while in eight cases the country of origin was not recorded. The notification rate among Syrians was almost seven times higher than that among the refugees from Afghanistan and Iraq. The distribution of cases and the notification rate per 1,000 estimated population by age group and country of origin is presented in Table 1.

**Table 1:** Number of notified cases and notification rate of hepatitis A in hosting facilities, by age group and country of origin, Greece, April–December 2016 (n=169^a^)

	Country of origin		
Age group (years)	Syria n (rate per 1,000)**^b^**	Afghanistan n (rate per 1,000)^b^	Iraq n (rate per 1,000)**^b^**
0–4	43 (8.8)	4(2.1)	2 (1.8)
5–9	55 (11.9)	1 (0.5)	3 (2.5)
10–14	32 (9.5)	2 (1.2)	4 (4.2)
15–29	22 (2.2)	1 (0.1)	0 (0.0)
**Total**	**152 (5.0)**	**8 (0.5)**	**9 (1.2)**

^a^ For eight cases the country of origin was not known.

^b^ Notification rate per 1,000 estimated population as described in the text.

One hundred and fifteen cases (65%) were notified from northern Greece, 44 (25%) from central Greece, 16 (9%) from Attica and 2 (1%) from eastern Aegean. In five camps the occurrence of HA cases lasted for several weeks and mass childhood vaccination was undertaken. Data regarding these camps are summarised in Table 2.

**Table 2:** Summary of data regarding the most affected camps and results of the mass and ring vaccination efforts, Greece, April–December 2016

Hosting camp	Number of recorded cases	Population^a^	Time^b^ (weeks)	Median age (years) (range: min–max)	Number of children aged 1–14 years vaccinated during mass vaccination	Number of close contacts aged 1–14 years vaccinated during ring vaccination	Number of close contacts aged 15 years or older vaccinated during ring vaccination	Proportion of hosted population vaccinated (%)
A	25	1,300	17–44	8 (1–29)	440	45	25	39
B	51	1,900	21–47	8 (2–27)	274	51	66	20
C	8	361	23–25	7.5 (3–17)	126	0	49	48
D	33	700	26–36	9 (1–28)	139	36	63	34
E	10	233	43–50	7 (2–12)	103	2	0	45
**Total**	**127**	**4,494**	**NA**	**7 (1–29)**	**1,082**	**134**	**203**	**32**

NA: not applicable.

^a^ Estimated average population living in the camps during reported HAV transmission.

^b^ Time between the first and the last reported case in the hosting camp.

During this period, four HA cases were reported via the mandatory notification system among staff responsible for cleaning the lavatories and other common areas in two hosting camps. These cases were hospitalised with jaundice and fever and were discharged from the hospital 8 to 10 days after full recovery. No other cases among the members of the non-governmental organisations or people working at or visiting the hosting facilities have been identified. No secondary community cases related to the cases in the hosting facilities have been recorded.

Based on the results from the Regional Laboratory of Public Health of Thessaly all seven samples tested were HAV genotype I subtype B.

### Management and control measures

A protocol for the management and response to the occurrence of HA cases at refugee hosting facilities was developed by the epidemiologists of HCDCP in early April as an adaptation of the ‘Hepatitis A management protocol for sporadic cases and outbreaks’, already available at HCDCP [5].

During intervention, focus was placed on hygiene measures and vaccination of close contacts of sporadic cases, within 14 days after their last contact with the case (ring vaccination). Priority was given to vaccination of children aged 1–14 years. For contacts aged 15 years old or older, serological testing for anti-HAV IgG and consequent vaccination according to result was recommended, given that the cost of testing was lower than the vaccination cost and that testing could be performed in time, without cancelling out the benefit from vaccination. If serological testing was not possible, vaccination was recommended instead. It should be noted that even though serological testing was included in the protocol, in practice, most adult contacts (aged 15 years or older) were vaccinated without prior serological testing because of time constrains.

Refugees were specifically advised on hygiene measures and the need for thorough hand washing with soap and water. In addition, brochures as well as posters with instructions on personal hygiene translated into Arabic, Urdu and Farsi were distributed to the population at hosting facilities. Hygiene rules regarding drinking water, food preparation and waste disposal were promoted in cooperation with the local public health authorities.

When cases occurred in a camp, staff and volunteers were also informed about the disease, the modes of transmission and the necessary hygiene measures.

According to the National Immunisation Programme, all cleaning staff and people working in waste and sewage management are advised to get vaccinated against HAV [6].

Vaccination of the entire childhood population was decided in five camps during this period, and performed with the co-operation of the non-governmental organisations (NGOs) (Table 1).

In total, 1,681 refugees were vaccinated from April to December 2016, with 1,082 (64.4%) vaccinations being part of the mass child vaccination at the five camps, and 599 vaccinations (of 309 and 290 contacts aged 1–14 years and 15 years old or older, respectively) performed during ring vaccination of the 177 reported cases.

## Hepatitis A notifications and situation of refugees in Greece

HA is a mandatory notifiable disease in Greece. Incidence in the general population, as well as the number of travel-related cases has been quite stable in recent years [7]. The mean notification rate for 2010–2015 was 0.72 per 10,000 population (SD: 0.39). In 2015 and the first trimester of 2016, 15 and 10 cases respectively were reported among refugees travelling via Greece to other European countries. During this time, it was estimated by the United Nations High Commission for Refugees (UNHCR) that there were 857,000 and 151,000 arrivals to Greece respectively, and most of these persons left the country after a few days’ stay [3].

In March 2016, the northern borders of Greece closed and Greece, from a previously mainly transit country, turned into one of medium-term stay and a large number of refugees were ‘stranded’ in the country [3]. The population residing in hosting facilities was estimated to be around 52,000 on 1 April; by 31 December, this number had increased to 62,700 people [1]. At the end of December, the hosting facilities included 51 camps (including first reception centres in the Aegean islands), and several hotels and apartments, under an UNHCR initiative [1]. The size and the demographic characteristics of the population residing in each of the hosting facilities have been changing in the period mentioned above because of the arrival of new refugees and their mobility inside the country. The majority of refugees who arrived to Greece during 2016 originated from Syria, Afghanistan, and Iraq [3].

## Discussion

HAV was frequently reported among refugees residing in hosting facilities in Greece from April to December 2016. Most cases were reported in children aged 1–14 years. Overcrowding and poor personal hygiene at hosting facilities are among the main predisposing factors for HAV infection in refugees. Children are the main pool of susceptible population, since adult refugees from HAV endemic countries are expected to be immune due to prior infection. In most instances, children are not vaccinated and often experience asymptomatic infection; thus, the disease can easily spread among them.

Genotyping of the virus infecting the reported cases showed that the virus belonged to hepatitis A subgenotype IB. Based on the literature, genotype I is more prevalent than other genotypes worldwide, and subtype A is more common than subtype B [8]. However, worldwide, in most regions, there is co-circulation of IA and IB strains but the predominant strain usually accounts for more than 95% of HAV strains. Based on the recorded data, in Turkey and Middle East, 95% of HAV strains belong to genotype I subtype B [9,10], whereas in Europe the predominant strain is genotype I subtype A [11]. In Greece, the available data are in accordance with the other European countries [12]. The findings presented may suggest the possible introduction of IB strains in Greece by refugees, and highlight the importance of molecular testing in mobile populations. Results, if provided in a timely fashion, would help understand transmission patterns and document introduction of possibly new hepatitis A strains in the European Union (EU). Molecular surveillance of cases in both refugees and the local population will continue.

The occurrence of HA mostly among Syrian children, implies higher susceptibility among them, compared with children from Afghanistan and Iraq, probably reflecting the epidemiology of the disease in the countries of origin.

In a 2000 study, anti-HAV IgG were present in 89% of the Syrian population with 50% in the 1–5 years age group and 95% in the 11–15 years age group [13]. Seroprevalence studies in the following years are not available and it is unknown whether there was a shift in HAV infection to an older age as in other neighbouring developing countries [14-16]. The notification rates by age group in the Syrian refugee population indicate that the seroprevalence of Syrian children and young adults might be lower than described in the literature and seroprevalence data for these age groups are needed. High incidence of HA among Syrian refugees has also been reported from Turkey [17], while a HA outbreak among Syrian refugees in northern Iraq has been documented [18]. Moreover, most children have asymptomatic HAV infection; thus, the number of infections that occurred during the study period was probably much higher than reported. An overestimated HAV seroprevalence contributed to the lack of routine HAV vaccination in Syrian refugee children, not only in Greece, but also in other countries hosting Syrian refugees.

The risk of occurrence of secondary cases in the community appears low based on the epidemiological data available so far.

Finally, even though the risk of disease transmission to volunteers and staff at the hosting facilities is small, we identified four cases among the cleaners at the most affected camps so advice has been given to the aforementioned population group.

Limitations of the case management varied widely depending on the characteristics and the living conditions at each hosting centre. Accommodation type (organised centre, hotel, apartment), size and demographics of the hosted population, nationalities, degree of organisation and activities of NGOs, substantially vary from one facility to another and management had to be tailored based on these specific characteristics.

Other factors, such as the proximity of the facility to the local hospital and the ability of the local authorities to take action to ensure the implementation of the hygiene measures at the camp that largely depends on the availability of human and other resources of the local authority, were also taken into account during response.

Response was challenging at prefectures with more than one established camp because of the potential competing priorities at a given time (e.g. in the event of HAV cases in more than one camps). Surge capacity issues were faced by local hospitals due to either: (i) hospitalisation of all HAV cases in order to reduce transmission rather than severity of disease and (ii) serologically testing of contacts before vaccination. Thus, other healthcare facilities were asked to support management of cases.

An additional challenge was the exact identification of close contacts, which was not easy in most of the cases. Family members and people living in the same room/tent were always offered vaccination, but it was particularly difficult to list all the other possible close contacts inside the hosting camp; the help of the medical staff/volunteers working in the area was of great importance in the early identification of contacts or additional cases.

Another obstacle in contact tracing was population mobility. There was constant movement of refugees from one facility to another and tracking down some of the contacts proved impossible in some occasions.

Finally, making vaccines available was time consuming, especially during national holidays; this led to delays in timely intervention in some instances.

At the moment, enhanced surveillance and timely vaccination of contacts is our main priority. Hygiene standards are necessary for preventing further occurrence of the disease. The ultimate goal is to have the entire refugee child population (1–14 years) fully follow the routine national childhood immunisation programme in Greece according to which, all children older than 12 months are vaccinated against HAV. The improvement of living conditions in hosting facilities will further limit the occurrence of HAV in this susceptible population.
